# The SMAC mimetic BV6 sensitizes colorectal cancer cells to ionizing radiation by interfering with DNA repair processes and enhancing apoptosis

**DOI:** 10.1186/s13014-015-0507-4

**Published:** 2015-09-17

**Authors:** Stephanie Hehlgans, Julius Oppermann, Sebastian Reichert, Simone Fulda, Claus Rödel, Franz Rödel

**Affiliations:** Department of Radiotherapy and Oncology, Goethe University Frankfurt, Theodor-Stern-Kai 7, 60590 Frankfurt am Main, Germany; Institute for Experimental Cancer Research in Pediatrics, Goethe University Frankfurt, Komturstr. 3a, 60528 Frankfurt am Main, Germany; German Cancer Research Center (DKFZ), Im Neuenheimer Feld 280, 69120 Heidelberg, Germany; German Cancer Consortium (DKTK) partner site: Frankfurt, Im Neuenheimer Feld 280, 69120 Heidelberg, Germany

## Abstract

**Background:**

In the present study, we aimed to investigate the effect of counteracting inhibitor of apoptosis (IAP) proteins using the small molecule Second Mitochondria-derived Activator of Caspase (SMAC) mimetic BV6 in combination with ionizing radiation on apoptosis, cell cycle regulation, DNA double-strand break (DSB) repair, three-dimensional (3D) clonogenic survival and expression of IAPs in colorectal carcinoma cells.

**Material and methods:**

Colorectal cancer cell lines (HCT-15, HT-29, SW480) were subjected to BV6 treatment (0–4 μM) with or without irradiation (2–8 Gy, single dose) followed by MTT, Caspase 3/7 activity, γH2AX/53BP1 foci assays, AnnexinV staining, cell cycle analysis, 3D colony forming assays and Western blotting (cellular IAP1 (cIAP1) and cIAP2, Survivin, X-linked IAP (XIAP)).

**Results:**

BV6 treatment decreased cell viability and significantly increased irradiation-induced apoptosis as analyzed by Caspase 3/7 activity, AnnexinV-positive and subG1 phase cells. While basal 3D clonogenic survival was decreased in a cell line-dependent manner, BV6 significantly enhanced cellular radiosensitivity of all cell lines in a concentration-dependent manner and increased the number of radiation-induced γH2AX/53BP1-positive foci. Western blot analysis revealed a markedly reduced cIAP1 expression at 4 h after BV6 treatment in all cell lines, a substantial reduction of XIAP expression in SW480 and HT-29 cells at 24 h and a slightly decreased cIAP2 expression in HCT-15 cells at 48 h after treatment. Moreover, single or double knockdown of cIAP1 and XIAP resulted in significantly increased residual γH2AX/53BP1-positive foci 24 h after 2 Gy and radiosensitization relative to control small interfering RNA (siRNA)-treated cells.

**Conclusion:**

The SMAC mimetic BV6 induced apoptosis and hampered DNA damage repair to radiosensitize 3D grown colorectal cancer cells. Our results demonstrate IAP targeting as a promising strategy to counteract radiation resistance of colorectal cancer cells.

**Electronic supplementary material:**

The online version of this article (doi:10.1186/s13014-015-0507-4) contains supplementary material, which is available to authorized users.

## Background

Colorectal carcinoma is the third most prevalent cancer and constitutes the fourth most common cause of cancer-related death worldwide [1]. Since publication of the first results of the CAO/ARO/AIO-94 study, preoperative radiochemotherapy provides the standard treatment of locally advanced rectal cancer [2, 3]. However, tumor cells frequently develop strategies to escape cell death upon radio- and/or chemotherapeutic treatment which interferes with efficient treatment of the patients. To overcome therapeutic limitations, efforts have been made to identify factors resulting in a therapy resistance and to target those factors, which may improve clinical outcome [4].

In this context, members of the inhibitor of apoptosis (IAP) protein family recently gained attention as attractive target molecules for sensitizing tumor cells to radiation therapy [5, 6]. Currently, eight different IAPs are known in mammals. Amongst them, Survivin has been extensively studied because of its multiple functions which comprise not only inhibition of Caspases and apoptosis but also regulation of cell division as part of the chromosomal passenger complex and radiation-induced damage repair [7–9]. Notably, overexpression of Survivin and a second well-studied member of this protein family, X-linked IAP (XIAP), is associated with a resistant phenotype in advanced rectal cancer after preoperative radiochemotherapy marked by increased local failure rates, distant metastasis and decreased overall survival [10, 11].

A common structural feature of IAPs is their baculovirus IAP repeat (BIR) domain, present in different numbers in all IAPs and required for apoptosis inhibition [12]. This structural domain is responsible for multiple protein interactions and regulation of IAP function. For Caspase inhibition, interaction of Survivin with XIAP by their BIR domains and with hepatitis B X-interacting protein (HBXIP) has been shown to be essential, while direct binding to Caspases 3, 7 and 9 is only mediated by XIAP [13, 14]. The carboxy-terminal Really Interesting New Gene (RING) domain, present for example in cellular IAP1 (cIAP1), cIAP2 and XIAP, functions as an E3 ubiquitin ligase and promotes ubiquitination and subsequent proteasomal degradation of the respective IAP and some of their binding partners [15, 16].

Amongst various IAP targeting approaches developed during the last years, substances mimicking the binding motif of the IAP antagonist Second Mitochondria-derived Activator of Caspase (SMAC) have gained growing attention. SMAC is released from mitochondria into the cytosol upon the induction of the intrinsic apoptosis pathway to negatively regulate IAP activity by binding to the BIR domains [17, 18]. The interaction between SMAC and XIAP, for example, prevents interaction of XIAP with Caspase 9 and subsequent activation of the apoptotic pathway [13]. Although the functions of cIAP1 and cIAP2 are less clear compared to XIAP and Survivin, it has been shown that both can function as E3 ubiquitin ligases and contribute to regulation of canonical and non-canonical nuclear factor kappa B (NF-κB) signaling pathways and are involved in the upregulation of cytotoxic cytokines like tumor necrosis factor-alpha (TNF-α) [15]. The latter renders human cancer cells susceptible to apoptosis induction in an autocrine/paracrine manner [19].

The bivalent SMAC mimetic BV6 binds to the BIR domains of IAP proteins, causing ubiquitination and proteasomal degradation of cIAPs and prevents XIAP-mediated Caspase inhibition leading to apoptosis induction as single agent treatment. Its therapeutic potential, however, is enhanced when combined with additional anticancer agents or ionizing irradiation [20–22]. In terms of combination with ionizing radiation, own previous data showed potent and concentration-dependent BV6-mediated radiosensitization of a panel of glioblastoma cell lines in a conventional two-dimensional (2D) colony formation assay [23].

In the present study, we aimed to examine cytotoxicity, apoptosis, cell cycle distribution, expression of IAPs Survivin, cIAP1, cIAP2 and XIAP and used the more physiologic three-dimensional (3D) clonogenic survival assay in three colorectal cancer cell lines, pretreated with BV6 followed by exposure to different doses of ionizing radiation. In the above mentioned studies the contribution of SMAC mimetics to apoptosis-induced cell death was the main focus, in the present study we also aimed to analyze the impact of BV6 on radiation-induced DNA damage repair by evaluation of γH2AX/53BP1-positive nuclear foci.

## Materials and methods

### Cell culture

Human colorectal carcinoma cell lines SW480, HT-29 and HCT-15 were selected according to their intrinsic radiation sensitivity and obtained from the American Type Culture Collection (LGC-Promochem, Wiesbaden, Germany). SW480 is homozygous for p53 mutation Arg-273→His and Pro-309→Ser. HT-29 cells also carry the homozygous mutation Arg-273→His, while the HCT-15 cell line is heterozygous for p53 mutation Pro-153→Ala [24–26]. Cells were cultured in DMEM (SW480; Life Technologies, Darmstadt, Germany), McCoy’s 5A modified (HT-29; Biochrom, Berlin, Germany) or RPMI medium (HCT-15; Life Technologies), supplemented with 10 % fetal calf serum (PAA, Cölbe, Germany) and 50 U/ml penicillin and 50 μg/ml streptomycin (Sigma-Aldrich, Munich, Germany) at 37 °C, 5 % CO_2_ and 95 % humidity.

### BV6 treatment and irradiation procedure

The bivalent SMAC mimetic BV6 was kindly provided by Genentech Inc. (South San Francisco, CA, USA) [15]. Cells were treated with 0.1–4 μM BV6 as indicated or with equivalent amounts of the solvent dimethyl sulfoxide (DMSO; AppliChem, Darmstadt, Germany) 4 h before irradiation. Irradiation with single doses of 2–8 Gy was performed using a linear accelerator (SL-15, Elekta, Crawley, UK) with 6 MeV/100 cm focus-surface distance and a dose rate of 4 Gy/min.

### MTT assay

Cells were plated at a density of 1 × 10^3^ cells per 200 μl in a 96-well microplate, grown for 24 h and subsequently treated with 1 or 4 μM BV6 or DMSO 4 h before irradiation with indicated doses. After an additional 24 h of incubation at 37 °C and 5 % CO_2_, 3-(4,5-Methylthiazol-2-yl)-2,5-diphenyl-tetrazolium bromide (MTT, Applichem) was added (20 μl/well of a 5 mg/ml solution in phosphate buffered saline (PBS, Life Technologies)) for 4 h. Solubilization of the converted purple formazan dye was accomplished by adding 50 μl of 0.01 N HCl/20 % SDS per well and incubation overnight at 37 °C. The reaction product was quantified by measuring the absorbance at 570 nm using a microplate reader (Wallac VICTOR 1420, PerkinElmer, Waltham, USA).

### Caspase 3/7 assay, AnnexinV detection of apoptosis and flow cytometric analysis

Cells were treated with BV6 (0.1–4 μM) or DMSO control 4 h before irradiation. At 24 h after irradiation, Caspase 3/7 activity, AnnexinV-positive cells or cell cycle distribution was measured to determine apoptosis induction. For quantification of Caspase 3/7 activity, a CASPASE GLO™-assay (Promega, Mannheim, Germany) was used according to the manufacturer’s instructions and luminescence was measured with a Wallac VICTOR microplate reader. For detection of AnnexinV-positive cells, cells were harvested, washed with PBS and stained with Annexin-V-Fluos labeling solution (Roche Diagnostics, Mannheim, Germany) according to the manufacturer’s protocol. For analysis of cells in subG1, G1, S or G2/M phase of cell cycle, non-irradiated cells were collected by trypsinization, washed with PBS, fixed and stained with a solution containing 40 μg/ml propidium iodide (Sigma-Aldrich) and 40 μg/ml RNaseA (Qiagen, Hilden, Germany). Quantification of AnnexinV-positive, subG1, G1, S and G2/M phase cells was performed with a FACSCalibur flow cytometer (Becton Dickinson, Heidelberg, Germany) and Cellquest Pro software (Becton Dickinson).

### Small interfering RNA (siRNA)-mediated knockdown of cIAP1 and XIAP

The human cIAP1-specific siRNA was obtained from Eurofins Genomics (Ebersberg, Germany): cIAP1 siRNA: 5′-GGCCAAGAGUUUGUUGAUtt-3′ (sense) [27]. The human XIAP siRNA was obtained from Applied Biosystems (Darmstadt, Germany): 5′- GCAGAUUUAUCAACGGCUUtt-3′, siRNA ID s1456 [28]. A non-specific negative control siRNA was obtained from Qiagen (Hilden, Germany). siRNA transfection was performed at a total concentration of 40 nM control siRNA. For single cIAP1 or XIAP knockdown conditions, 20 nM specific siRNA was complemented with 20 nM control siRNA to achieve a total siRNA concentration of 40 nM. Double knockdown experiments were carried out using 20 nM cIAP1 and 20 nM XIAP siRNA. Cells were transfected with Roti-Fect PLUS reagent according to the manufacturer’s instructions (Roth, Karlsruhe, Germany).

### Immunofluorescence staining and quantification of γH2AX and 53BP1 foci formation

DNA double-strand breaks (DSB) were analyzed by counting of γH2AX- and 53BP1-positive nuclear foci as previously described [29]. In brief, colorectal carcinoma cells were cultured on 8-well slides (BD Falcon, Heidelberg, Germany), siRNA-transfected and irradiated 24 h thereafter or treated with DMSO, 1 μM or 4 μM BV6 and irradiated 4 h thereafter with a dose of 2 Gy. Subsequently, cells were fixed after indicated time points with either ice-cold methanol in case of γH2AX detection or 3.7 % paraformaldehyde in case of 53BP1 detection. Permeabilization was performed by addition of 0.1 % of Triton X-100, followed by blocking with 5 % BSA, 0.05 % Triton X-100, 1 μg/ml rabbit/mouse serum and incubation with anti-γH2AX (clone JBW301, 05–636, Millipore, Schwalbach, Germany) or anti-53BP1 (100–304, Novus Biologicals, Cambridge, UK) primary antibodies. Staining was accomplished by incubation with appropriate Alexa-labeled secondary antibodies (Alexa Fluor 594 goat anti-mouse, Alexa Fluor 488 goat anti-rabbit, Life Technologies) and counterstaining of nuclei with 4′,6-diamidino-2-phenylindole (DAPI) solution (Life Technologies). Coverslips were mounted with Vectashield mounting medium (Alexis, Grünberg, Germany). γH2AX- and 53BP1-positive foci were microscopically counted using an AxioImager Z1 microscope equipped with AxioVision 4.6 software (Carl Zeiss, Göttingen, Germany) and 100–150 nuclei were evaluated for each data point from three independent experiments. Fluorescence images were obtained using the AxioImager Z1 microscope equipped with AxioVision 4.6 software (Carl Zeiss).

### 3D colony formation assay

Measurement of 3D cell survival was accomplished as described before [30, 31]. In brief, single cells were plated into a mixture of 0.5 mg/ml laminin-rich extracellular matrix (lrECM; Cultrex 3D Culture Matrix BME Reduced Growth Factor Basement Membrane Extract; R&D Systems, Wiesbaden, Germany) in 96-well plates. After 24 h, cells were treated with DMSO, 0.1, 0.25, 1 or 1.5 μM BV6 or left untreated (mock) and irradiated (2, 4, 6 Gy) 4 h later. For cIAP1 and/or XIAP knockdown experiments, cells were transfected with siRNA and plated 24 h thereafter into lrECM 3D matrix. Irradiation of cells with single doses of 0, 2, 4, 6 Gy was performed 24 h after plating. Colonies (>50 cells) were microscopically counted at least 6 days after plating, dependent on the cell line used. Images from typical colony formation of colorectal cancer cell lines were obtained using an AxioImager Z1 microscope. Surviving fractions from 3D clonogenic assays were calculated as follows: numbers of colonies formed/(numbers of cells plated (irradiated) × plating efficiency (non-irradiated)). Each point on survival curves represents the mean surviving fraction from at least three independent experiments, each performed in quadruplicate. Survival variables α and β were fitted according to the linear quadratic equation (SF = exp [−α × D − β × D^2^] with D = dose using EXCEL software (Microsoft, Redmond, USA).

### Western blotting

Cells were plated in 0.5 mg/ml lrECM for 24 h, treated either with DMSO or 1 μM BV6 4 h before irradiation with 4 Gy and subjected to Western blotting at different time points thereafter as reported before [30, 31]. The following primary antibodies were applied: anti-cIAP1 (AF8181, R&D Systems), anti-cIAP2 (1040–1, Abcam, Cambridge, UK), anti-Survivin (AF886, R&D Systems), anti-XIAP (BD610716, Becton Dickinson) and anti-β-actin (A5441, Sigma-Aldrich). For detection, secondary antibodies chicken anti-goat IgG HRP (sc-2953; Santa Cruz, Heidelberg, Germany), goat anti-rabbit IgG HRP (sc-2054; Santa Cruz), goat anti-mouse IgG HRP (sc-20559; Santa Cruz), Pierce ECL Western Blotting Substrate and Amersham Hyperfilm ECL (Thermo Fisher Scientific, Waltham, USA) were used.

### Data analysis

To test statistical significance, the two-sided unpaired Student’s *t*-test was performed using EXCEL software (Microsoft, Unterschleißheim, Germany). Results were considered statistically significant if a *p*-value of less than 0.05 was reached.

## Results

**Fig. 1:**
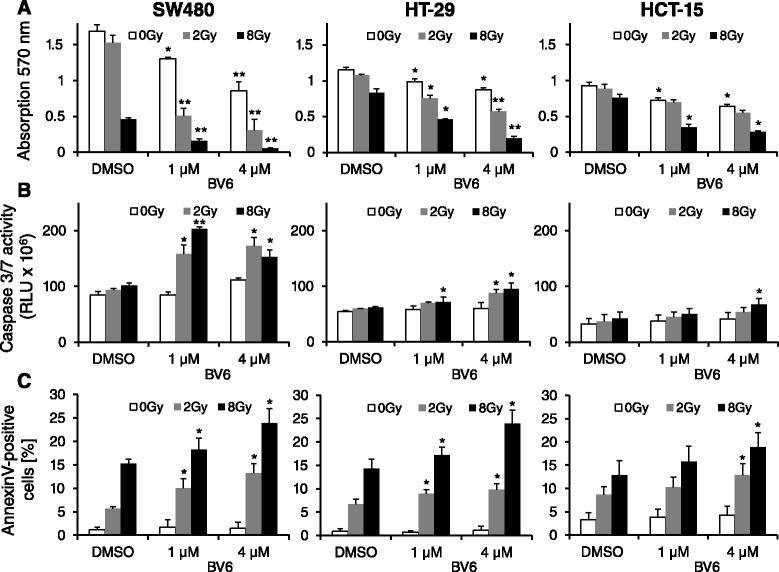
Combined BV6 treatment and irradiation decreases cell viability and enhances apoptosis of colorectal carcinoma cells. SW480, HT-29 and HCT-15 cells were treated with 1 μM or 4 μM BV6 or with DMSO as a control for 4 h and were subsequently irradiated with a dose of 0, 2 or 8 Gy. After 24 h, cells were subjected to a colorimetric MTT-assay (**a**), analysis of Caspase 3/7 activity (**b**) or flow cytometric measurement of AnnexinV-positive cells (**c**). Results represent means ± SD (*n* = 3). Student’s *t*-test compared BV6-treated vs. DMSO-treated cells (*P <0.05; **P <0.01)

**Fig. 2:**
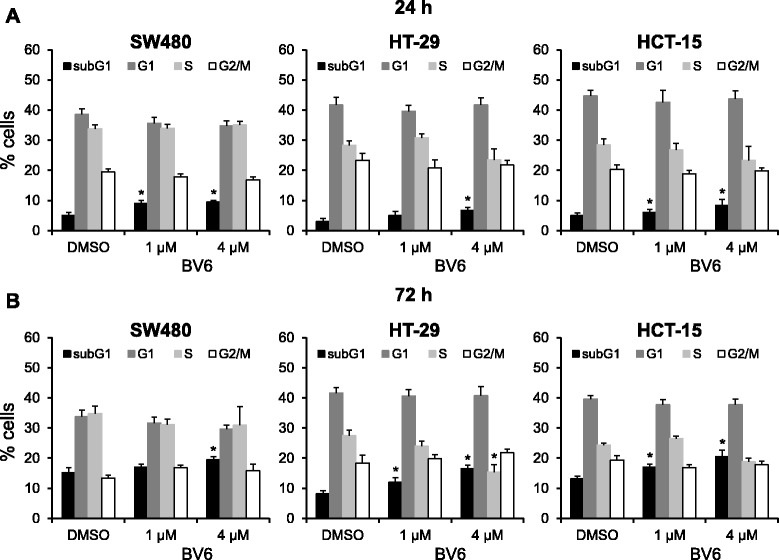
BV6 increases the fraction of colorectal cancer cells in subG1 phase. **a** – **b** Flow cytometric analysis of subG1, G1, S and G2/M phase colorectal cancer cells at indicated time points after treatment with BV6 or DMSO control. Data are displayed as means ± SD from three independent experiments. Asterisks indicate significant differences (*P <0.05; Student’s *t*-test) as compared to DMSO-treated controls

To investigate potential cytotoxic effects of the SMAC mimetic BV6 alone or in combination with irradiation, a colorimetric MTT assay was applied. As shown in Fig. 1a, a 24 h BV6 treatment reduced SW480, HT-29 and HCT-15 colorectal cancer cell viability in a concentration- and cell line-dependent manner with a significant reduction of viability following sole BV6 treatment. This effect was further enhanced by additional irradiation (2 or 8 Gy) at 4 h after BV6 treatment, most pronounced in SW480 and HT-29 cells (Fig. 1a). Next, the impact of BV6 and irradiation on apoptosis induction was evaluated by measurement of Caspase 3/7 activity, detection of AnnexinV-positive cells (Fig. 1) and determination of subG1 apoptotic cells (Fig. 2). Treatment with BV6 substantially increased Caspase 3/7 activity (Fig. 1b) and the fraction of AnnexinV-positive cells (Fig. 1c) 24 h after irradiation with a dose of 2 or 8 Gy. Moreover, BV6 application resulted in a significant increase of the subG1 population of non-irradiated cells at 24 h (Fig. 2a) and 72 h (Fig. 2b) post BV6 treatment, while cell cycle distribution and the fraction of cells in G1, S or G2/M phase were not substantially altered by treatment with the SMAC mimetic (Fig. 2a, b).

**Fig. 3:**
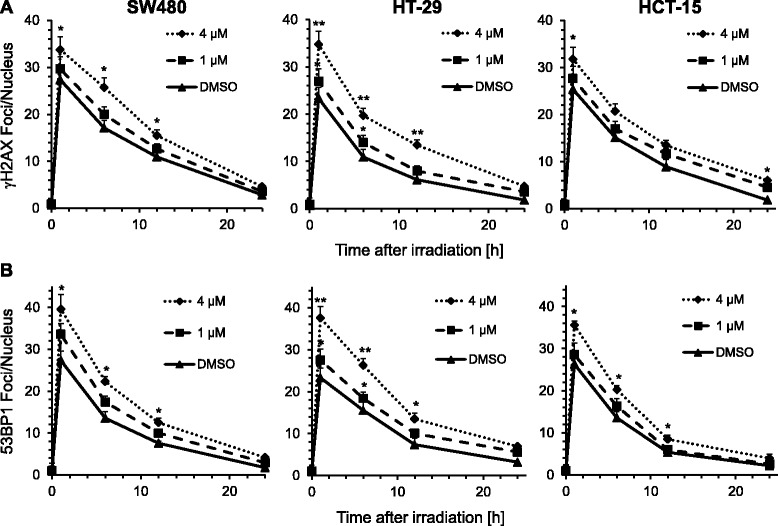
Treatment with BV6 elevates irradiation-induced nuclear foci formation. SW480, HT-29 and HCT-15 colorectal carcinoma cell lines were treated either with DMSO as a control or with 1 or 4 μM BV6 4 h before irradiation. Immunofluorescence staining for γH2AX (**a**) or 53BP1 (**b**) was accomplished 0, 1, 6, 12 and 24 h after irradiation with 2 Gy and nuclear foci were counted subsequently (means ± SD; *n* = 3; * *P* <0.05; Student’s *t*-test)

To investigate a potential impact of the drug on DNA repair capacity, cells were next treated for 4 h with 1.0 or 4.0 μM BV6, irradiated with a dose of 2 Gy and subjected to γH2AX or 53BP1 foci analysis at 0, 1, 6, 12 or 24 h after irradiation. Both, γH2AX and 53BP1 radiation-induced nuclear foci were significantly increased after 4 μM BV6 treatment as compared to DMSO controls, suggesting a BV6-dependent modulation of DNA repair pathways in SW480, HT-29 and HCT-15 colorectal cells (Fig. 3a, b and Additional file 1: Figure S1).

**Fig. 4:**
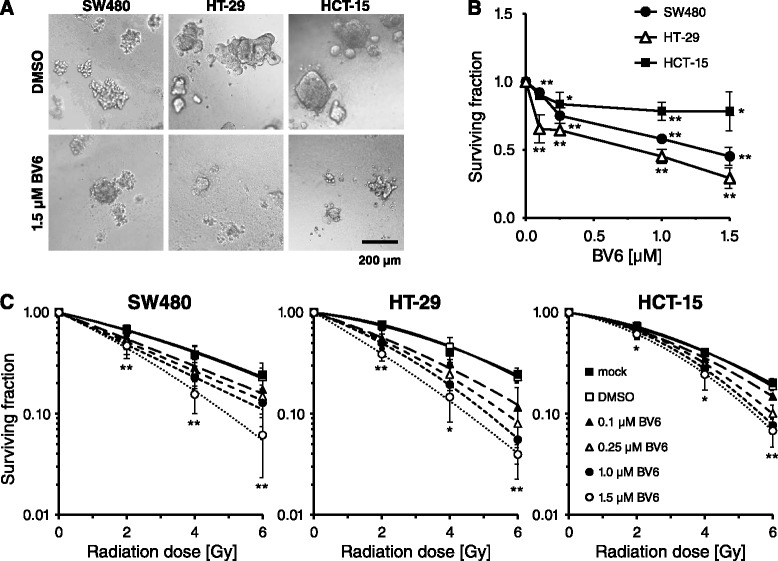
BV6 treatment decreases basal clonogenic survival and substantially radiosensitizes colorectal cancer cells. Single cells were plated in a 3D extracellular matrix and 24 h thereafter, cells were treated either with DMSO or increasing concentrations of BV6 (0.1, 0.25, 1, 1.5 μM) or were left untreated (mock). At 4 h after BV6 treatment, cells were irradiated with 0, 2, 4 or 6 Gy (single doses) and ≥6 days after plating colonies >50 cells were microscopically counted. **a** Typical colony formation of DMSO- or BV6-treated colorectal cancer cell lines ≥6 days after plating (bar, 200 μm). **b** Survival curves of indicated colorectal cancer cell lines after BV6 treatment were calculated and normalized to DMSO-treated cells (means ± SD; *n* ≥3; *P <0.05; **P <0.01; Student’s *t*-test). **c** To evaluate 3D radiation survival, single cells were pretreated for 4 h with DMSO or increasing concentrations of BV6 or left untreated (mock). Subsequently, cells were irradiated (0–6 Gy, single dose) and survival fractions relative to non-irradiated controls were calculated. Curves were fitted according to the linear quadratic model (means ± SD; *n* ≥3; *P <0.05; **P <0.01; Student’s *t*-test in comparison to DMSO-treated controls)

For analysis of long term clonogenic survival, cells were plated in a more physiologic 3D laminin-rich extracellular matrix, treated with 0.1, 0.25, 1.0 or 1.5 μM BV6 and clonogenic survival was microscopically evaluated 6–8 days thereafter (Fig. 4a). Basal clonogenicity was concentration- and cell line-dependently decreased with the most pronounced effect on HT-29 cells, demonstrated by a 70.8 % reduced survival after treatment with 1.5 μM BV6. In contrast, HCT-15 responded only mildly to BV6 treatment, while treatment of SW480 cells with 1.5 μM BV6 resulted in a medium response, illustrated by a 54.8 % reduction of clonogenic survival (Fig. 4b). When combined with ionizing radiation, BV6 pretreatment resulted in a concentration-dependent radiosensitization in all three lines investigated, again most pronounced in HT-29 cells (Fig. 4c).

**Fig. 5:**
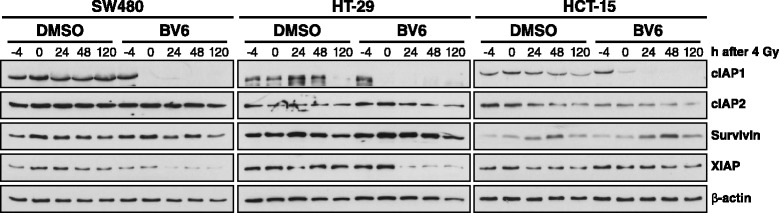
BV6 considerably reduces expression of IAP proteins. SW480, HT-29 and HCT-15 cells were plated in a 3D laminin-rich extracellular matrix and treated with 1 μM BV6 or DMSO control 4 h before irradiation with 4 Gy. At different time points, expression of indicated proteins was analyzed by Western blotting, while β-actin served as loading control. Two independent experiments were performed with similar results

To test the effect of the SMAC mimetic in combination with irradiation on the expression of several IAPs, Western blot analyses were applied. As depicted in Fig. 5, we observed a cell line-dependent degradation of IAP proteins after BV6 exposure, while irradiation does only marginally modulate cIAP1, cIAP2 or XIAP expression. In detail, a treatment of SW480, HT-29 and HCT-15 cells with 1 μM BV6 resulted in a rapid degradation of cIAP1 with non-detectable levels after a 4 h treatment in SW480 and HT-29 cells. XIAP was degraded at 24 h after BV6 exposure in SW480 and HT-29 cells, while HCT-15 cells showed no effect. By contrast, cIAP2 and Survivin expression were only slightly affected by BV6 treatment, with a moderate decreased expression at 48 h (cIAP2) and 120 h (cIAP2 and Survivin) in all cell lines.

**Fig. 6:**
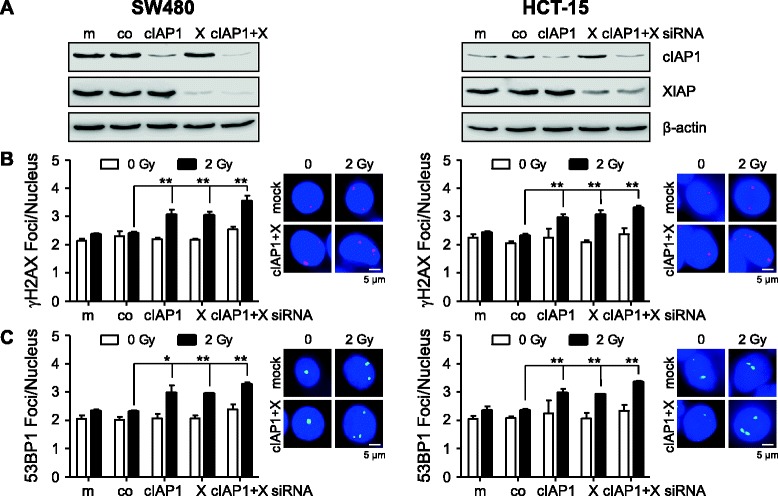
cIAP1 and XIAP depletion hampers DNA double-strand break repair. SW480, and HCT-15 colorectal cancer cells were subjected to siRNA-mediated cIAP1 and/or XIAP knockdown 48 h before irradiation. **a** Western Blot analysis confirmed knockdown of cIAP1 and XIAP (X) at 48 h after siRNA transfection, while β-actin served as loading control. Immunofluorescence staining for γH2AX (**b**) or 53BP1 (**c**) was accomplished 24 h after irradiation with 2 Gy and nuclear foci were counted subsequently (means ± SD; *n* = 3; * *P* <0.05; **P <0.01; Student’s *t*-test). Representative photographs of indicated conditions show staining of nuclei in blue (DAPI), γH2AX in red and 53BP1 in green (scale bar, 5 μm)

**Fig. 7:**
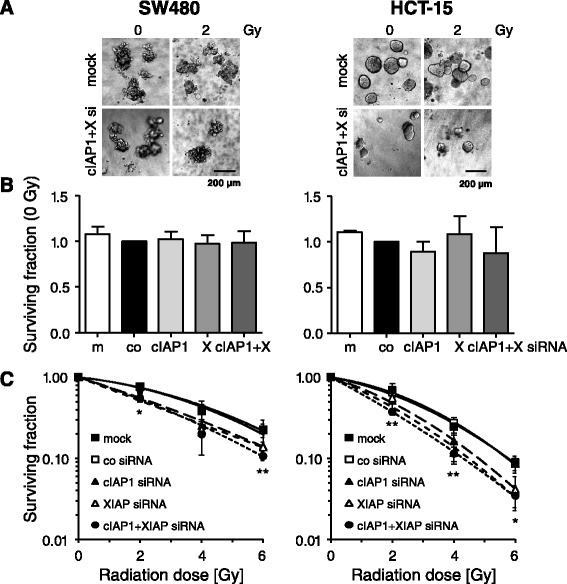
cIAP1 and XIAP knockdown radiosensitizes colorectal cancer cells. Single cells from knockdown cell cultures were plated in a 3D extracellular matrix and irradiated with 0, 2, 4 or 6 Gy (single doses) 24 h thereafter. Colonies with more than 50 cells were counted microscopically 6–7 days after plating. **a** Typical 3D colony formation of non-irradiated (0 Gy) or irradiated (2 Gy) mock-treated (mock) or cIAP1/XIAP depleted (cIAP1+X si) cell cultures 6–7 days after plating (bar, 200 μm). **b** Plating efficiency of 3D grown mock, control siRNA (co), cIAP1 siRNA (cIAP1), XIAP siRNA (X) and cIAP1/XIAP (cIAP1+X) siRNA transfected cells was determined and normalized to control siRNA (co) treated cells. (means ± SD; *n* ≥3). **c** For measurement of 3D radiation survival, indicated knockdown cell cultures were irradiated (0–6 Gy, single dose) and survival fractions relative to non-irradiated controls were calculated. Curves were fitted according to the linear quadratic model (means ± SD; *n* ≥3; *P <0.05; **P <0.01; Student’s *t*-test in comparison to control (co) siRNA treatment)

To further confirm that a degradation of cIAP1 and XIAP is correlated to a hampered DNA repair and clonogenic survival upon irradiation, we performed siRNA-mediated knockdown of cIAP1 and/or XIAP in SW480 and HCT-15 cells (Fig. 6a). Irradiation of single cIAP1 or XIAP knockdown cell cultures significantly increased residual γH2AX- and 53BP1-positive foci 24 h after 2 Gy relative to control siRNA-treated cells (Fig. 6b, c). Double knockdown of cIAP1 and and XIAP, however, only marginally further increased the number of residual γH2AX and 53BP1 foci in both SW480 and HCT-15 cells, indicating the involvement of common molecular pathways. In line with that, 3D radiation survival was significantly reduced after cIAP1, XIAP and cIAP1/XIAP depletion (Fig. 7a, c). The radiosensitization was most pronounced following a cIAP1 and XIAP double knockdown while basal clonogenic survival of non-irradiated cells was not altered by different knockdown conditions (Fig. 7b).

## Discussion

Evasion of apoptosis is a hallmark of cancer that contributes to tumorigenesis and tumor progression and displays a major obstacle in the efficacy of anticancer treatment such as radiation therapy [32]. Thus, interfering with the apoptotic pathways by the development of small molecule inhibitors that mimic the endogenous IAP inhibitory protein SMAC may present a promising strategy to counteract resistance. In the current study, we investigated the effect of combined BV6 SMAC mimetic treatment and ionizing radiation on cell viability, apoptosis, DNA repair and 3D clonogenic survival of three colorectal cancer cell lines. We found significantly enhanced effects in terms of viability, apoptosis induction, clonogenic survival and DNA damage repair after combined modality treatment with BV6 and irradiation.

There is growing knowledge on the mechanisms of a SMAC mimetic-mediated tumor cell sensitization that include increased caspase activation and engagement of an autocrine cell death loop via NF-κB mediated TNF-α/TNF receptor signaling [15, 19]. SMAC mimetics may further sensitize tumor cells to DNA damaging agents by favouring the assembly of a cytosolic multi-protein complex (Ripoptosome) containing receptor-interacting serine/threonine protein kinase 1 (RIP1). This complex signals cell death by stimulating NF-κB activation and induction of a non-apoptotic form of programmed cell death called necroptosis [33, 34]. Although not addressed in the present study, radiation sensitization upon BV6 treatment in colorectal cancer lines may arise from multiple mechanisms including apoptosis, necroptosis and mitotic catastrophe.

Indeed, radiation-induced cell death involves Caspase-dependent and Caspase-independent mechanisms [35]. Especially in solid tumors, mitotic catastrophe upon irreparable DNA damage is thought to constitute a mechanism contributing to cell inactivation. Mitotic catastrophe often occurs as a consequence of non-repaired DNA damage and a deficiency in cell cycle checkpoints and frequently results from the presence of mutated or inactivated p53 protein in tumor cells [36], which is the case in the cell lines investigated in the present study. Notably, radiation induced apoptosis upon XIAP knockdown is more pronounced in p53 mutated lung carcinoma cells, indicating that XIAP-mediated sensitization might be more effective in p53 mutated cells [37].

Since radiation sensitivity of cells varies considerably in dependence of their cell cycle phase [38], we analyzed the impact of BV6 on cell cycle distribution in non-irradiated cells. While BV6 treatment increased the fraction of cells in subG1 phase indicative of apoptosis in a time- and concentration-dependent manner (Fig. 2), we did not observe a significant impact of the BV6 mono substance on the number of cells in G1, S or in G2/M phase, suggesting that cell cycle regulation by BV6 treatment at the time of irradiation might not contribute to increased radiation response. Similar to our results, single BV6 treatment of Panc1 pancreatic carcinoma cells did not result in significant changes of G1, S or G2/M phases of the cell cycle [39].

Concerning the BV6- and irradiation-mediated modulation of IAP expression, we found a rapid degradation of cIAP1 in all three cell lines and a slower decrease of XIAP expression in two out of three cell lines. cIAP2 and Survivin expression, by contrast, were only moderately decreased in all lines at 120 h after BV6 treatment. Our observations are partly in line with previously published studies, revealing rapid degradation of cIAP1 and cIAP2 and slower degradation of XIAP in MDA-MB-231 breast carcinoma cells [15] or downregulation of cIAP1, cIAP2 and XIAP levels in MV4-11, OCI-AML3 and NB4 acute myeloid leukemia cells [20, 22] upon BV6 treatment. However, variances in cIAP2 degradation kinetics might result from the use of different cell lines, BV6 concentrations, time points and/or cell culture conditions (3D vs. 2D) used. Interestingly, SM-164 compound was shown to decrease cIAP1 expression and to prevent XIAP binding to caspases without affecting XIAP expression [40], further stressing the point on a cancer line specific regulation of IAPs upon SMAC mimetic treatment.

The impact of BV6 on clonogenic radiation survival was evaluated under 3D cell culture conditions, which are thought to mimic the *in vivo* situation more closely than the conventional 2D colony formation assays [41, 42]. BV6 reduced basal clonogenic survival of non-irradiated 3D cultured cells and increased radiation sensitivity of all three lines investigated (Fig. 4). The most prominent radiation sensitization effect was observed in HT-29 cells which nicely corresponded to the pronounced increased numbers of DNA DSBs upon BV6 treatment. Apoptosis induction was most prominent in SW480 cells. In line with that, studies conducted by Qin et al. and Huerta et al. reported on radiosensitization of esophageal carcinoma cells by the compound LCL161 [43], while the compound JP-1201 was shown to sensitize HT-29 colorectal carcinoma cells *in vitro* and in a SCID mouse xenograft model in conjunction with enhanced Poly(ADP-ribose) polymerase-1 (PARP-1) cleavage [43]. Notably, these studies also analyzed the impact of SMAC mimetic treatment on DNA damage response showing elevated numbers of radiation-induced γH2AX-positive nuclear foci. These results thus support our observations on a hampered DNA repair mediated by SMAC mimetics. Previous own data further revealed 3D radiosensitization in a panel of colorectal cancer cells upon siRNA-mediated attenuation of XIAP and/or Survivin [28] in line with an increased γH2AX foci detection following single XIAP and Survivin or double knockdown (unpublished results). To confirm the involvement of cIAP1 and XIAP in the modulation of DNA damage response, we here analyzed SW480 and HCT-15 radiation responses in the presence of siRNA targeting these proteins (Fig. 6). Irradiation of single cIAP1 or XIAP knockdown cells significantly increased γH2AX- and 53BP1-positive foci at 24 h while double knockdown of cIAP1 and XIAP only marginally further increased the number of residual foci. These data support our hypothesis of the involvement of IAPs in DNA DSB repair mechanisms, which has been proposed for Survivin before [29, 44]. Mechanistically, Survivin physically interacts with members of the DNA repair machinery, including DNA-dependent protein kinase, catalytic subunit (DNA-PK_CS_) and regulates DNA PKcs catalytic activity in SW480 colorectal cancer cells [29]. Here, we observed a hampered DNA damage repair upon SMAC mimetic-induced degradation of cIAP1 and XIAP, while Survivin expression was not significantly affected (Fig. 5). These results suggest an impact of additional members of the IAP family on DNA damage repair, stressing the significance of anti-IAP strategies to improve radiation therapy outcome. The underlying mechanism(s) on a cIAP1- and XIAP-mediated modulation of radiation-induced DSB repair, however, remain elusive and require further investigations.

In summary, our findings support the notion that BV6 mediated degradation of XIAP and cIAP1 results in radiosensitization of colorectal cancer cells via increased apoptosis and impaired DNA DSB repair.

## Conclusion

Intervention of IAP function with SMAC mimetic compounds emerges as a promising strategy to enhance radiation sensitivity of cancer cells. In the present study, the SMAC mimetic BV6 substantially increased 3D radiation response of three human colorectal cancer cell lines by degradation of cIAP1 and XIAP, thereby enhancing irradiation-induced apoptosis and hampering DNA DSB repair. Thus, BV6 may represent a molecular compound to improve multimodal therapeutic outcome of colorectal cancer.

## Additional file

Additional file 1: Figure S1. Treatment with BV6 increased radiation-induced DNA damage. SW480, HT-29 and HCT-15 colorectal carcinoma cell lines were treated either with DMSO as a control or with 4 μM BV6 4 h before irradiation. Immunofluorescence staining for γH2AX in red **(A)** or 53BP1 in green **(B)** was accomplished 24 h after irradiation with 0/2 Gy and nuclei were counterstained with DAPI. Photographs show representative images of nuclear foci of indicated conditions obtained with an AxioImager Z1 microscope equipped with AxioVision 4.6 software (Carl Zeiss). Scale bar, 5 μm. (PPT 4679 kb)
