# Supplementary material for: The SMAC mimetic BV6 sensitizes colorectal cancer cells to ionizing radiation by interfering with DNA repair processes and enhancing apoptosis
Source: Radiat Oncol. 2015 Sep 17;10:198. doi: 10.1186/s13014-015-0507-4 (PMC4573682; doi:10.1186/s13014-015-0507-4)
Supplement: Additional file 1: Figure S1. — Treatment with BV6 increased radiation-induced DNA damage. SW480, HT-29 and HCT-15 colorectal carcinoma cell lines were treated either with DMSO as a control or with 4 μM BV6 4 h before irradiation. Immunofluorescence staining for γH2AX in red (A) or 53BP1 in green (B) was accomplished 24 h after irradiation with 0/2 Gy and nuclei were counterstained with DAPI. Photographs show representative images of nuclear foci of indicated conditions obtained with an AxioImager Z1 microscope equipped with AxioVision 4.6 software (Carl Zeiss). Scale bar, 5 μm. (PPT 4679 kb) [file 13014_2015_507_MOESM1_ESM.ppt]

## Slide 1
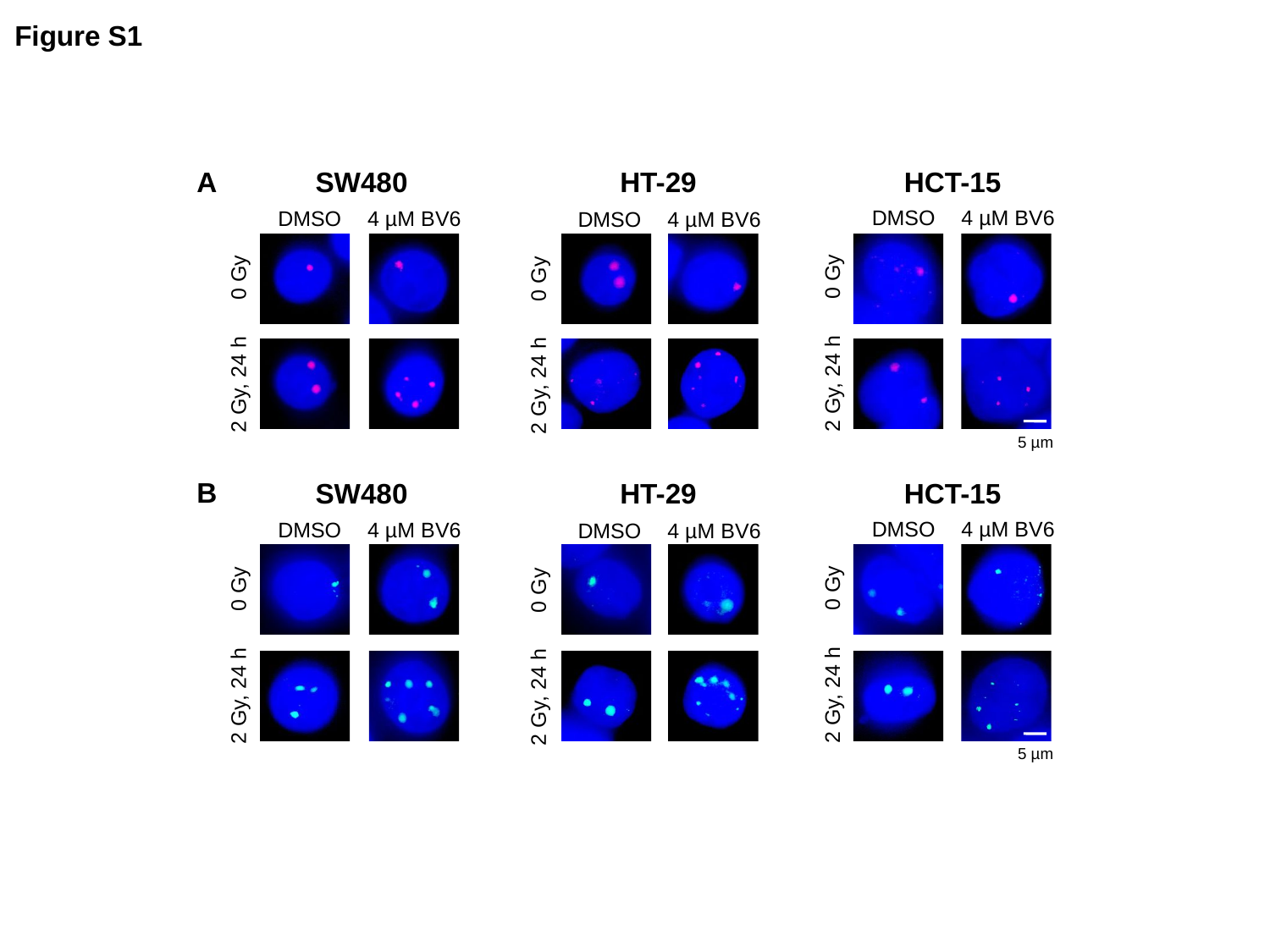

Figure S1
A
SW480
HT-29
HCT-15
DMSO
4 µM BV6
DMSO
4 µM BV6
DMSO
4 µM BV6
0 Gy
0 Gy
0 Gy
2 Gy, 24 h
2 Gy, 24 h
2 Gy, 24 h
5 µm
B
SW480
HT-29
HCT-15
DMSO
4 µM BV6
DMSO
4 µM BV6
DMSO
4 µM BV6
0 Gy
0 Gy
0 Gy
2 Gy, 24 h
2 Gy, 24 h
2 Gy, 24 h
5 µm
